# Transarticular screw fixation for atlantoaxial instability - modified Magerl's technique in 38 patients

**DOI:** 10.1186/1749-799X-5-87

**Published:** 2010-11-22

**Authors:** Raj Bahadur, Tarun Goyal, Saravdeep S Dhatt, Sujit K Tripathy

**Affiliations:** 1 Postgraduate Institute of Medical Education and Research, Chandigarh, India; 2 Government Medical College and Hospital, Chandigarh, India; 3 Dept of Orthopaedics, All India Institute of Medical Sciences, New Delhi, India; 4 Dept of Orthopaedics, Postgraduate Institute of Medical Education and Research, Chandigarh, India

## Abstract

**Background:**

Symptomatic atlantoaxial instability needs stabilization of the atlantoaxial joint. Among the various techniques described in literature for the fixation of atlantoaxial joint, Magerl's technique of transarticular screw fixation remains the gold standard. Traditionally this technique combines placement of transarticular screws and posterior wiring construct. The aim of this study is to evaluate clinical and radiological outcomes in subjects of atlantoaxial instability who were operated using transarticular screws and iliac crest bone graft, without the use of sublaminar wiring (a modification of Magerl's technique).

**Methods:**

We evaluated retrospectively 38 subjects with atlantoaxial instability who were operated at our institute using transarticular screw fixation. The subjects were followed up for pain, fusion rates, neurological status and radiographic outcomes. Final outcome was graded both subjectively and objectively, using the scoring system given by Grob et al.

**Results:**

Instability in 34 subjects was secondary to trauma, in 3 due to rheumatoid arthritis and 1 had tuberculosis. Neurological deficit was present in 17 subjects. Most common presenting symptom was neck pain, present in 35 of the 38 subjects.

Postoperatively residual neck and occipital pain was present in 8 subjects. Neurological deficit persisted in only 7 subjects. Vertebral artery injury was seen in 3 subjects. None of these subjects had any sign of neurological deficit or vertebral insufficiency. Three cases had nonunion. At the latest follow up, subjectively, 24 subjects had good result, 6 had fair and 8 had bad result. On objective grading, 24 had good result, 11 had fair and 3 had bad result. The mean follow up duration was 41 months.

**Conclusions:**

Transarticular screw fixation is an excellent technique for fusion of the atlantoaxial complex. It provides highest fusion rates, and is particularly important in subjects at risk for nonunion. Omitting the posterior wiring construct that has been used along with the bone graft in the traditional Magerl' s technique achieves equally good fusion rates and is an important modification, thereby avoiding the complications of sublaminar wire passage.

## Background

Atlantoaxial articulation is the most unique part of the spine. It is the most mobile segment of the spine, and largely depends on the ligamentous supports and the integrity of the odontoid for its stability. Fusion of the C1-C2 complex may be required in cases of atlantoaxial instability. Its extreme mobility places heavy demand on the atlantoaxial fixation construct for sufficient rigidity required for its fusion. The causes of C1-C2 instability are numerous and include trauma, congenital malformations, inflammatory arthritis, malignancies, skeletal dysplasias, rotatory subluxations and pharyngeal infections. Symptoms of instability of the atlanto axial complex are varied, such as neck pain, transient paresis, headaches, ataxia and intermittent loss of consciousness.

Clinically or radiographically significant atlantoaxial subluxation is best treated by reduction and fusion of the C1-C2 joint. Posterior C1-C2 fusion using transarticular screw (TAS), introduced by Magerl et al in 1979 [1] is the gold standard for atlantoaxial arthrodesis. It has the advantage of a more rigid fixation with higher rates of fusion, avoiding need for postoperative halo, no placement of implant in the spinal canal, and possibility of its use in anomalies of odontoid process or the posterior arch [2-7]. Magerl et al used two transarticular screws along with bone graft and interspinous wiring for fusion. But the use of sublaminar wiring is fraught with several complications, such as damage to the dura and the cord during insertion of the wires and late compression of the cord by wire breakage or loosening [8-12]. Further, it has been found that there may be no important contribution of the wires in holding the graft for fusion, and comparable fusion rates have been achieved in these studies [4,13,14].

Thus we designed our study to evaluate the outcome of cases of atlantoaxial instability treated with transarticular screw fixation. We did not include supplemental wiring as described by Magerl et al in our technique. Postoperatively, the subjects were evaluated clinically and radiographically for the improvement in clinical scores, fusion rates of the arthrodesis and any associated complications.

## Methods

**Figure 1:**
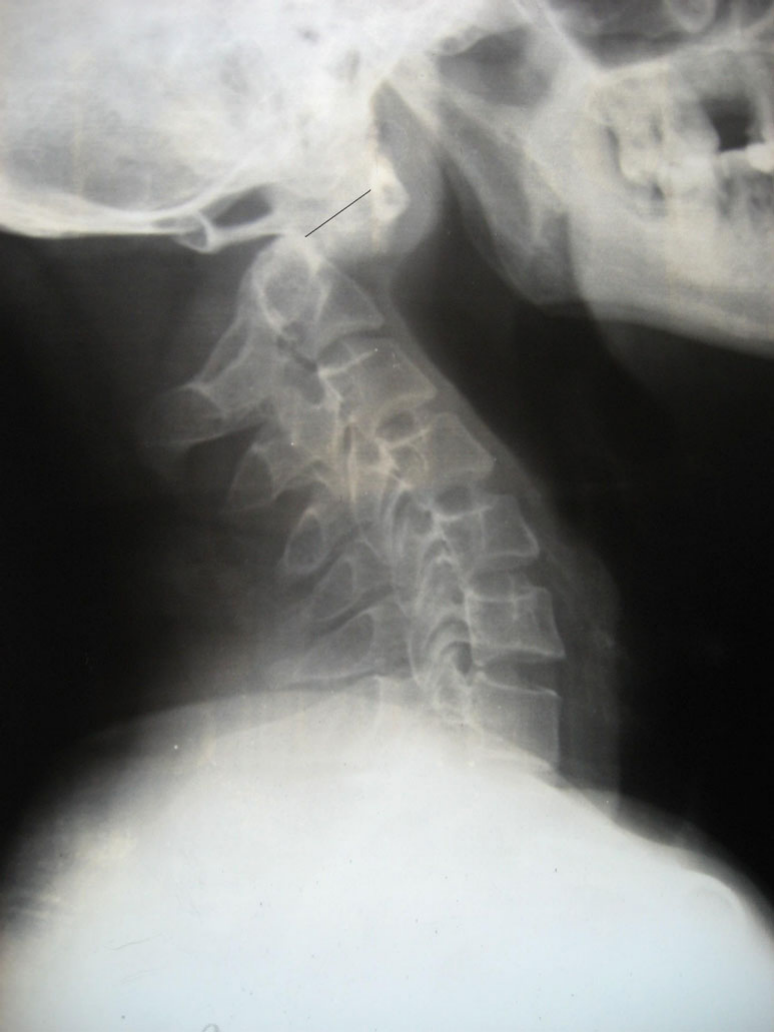
**Lateral radiograph of a subject with atlantoaxial instability secondary to odontoid fracture showing marked atlantoaxial displacement**.

We studied 38 subjects of atlantoaxial instability who underwent posterior fusion using transarticular screws. All the cases were operated by the senior author (RB) from 1995 to 2008. Instability was defined on flexion-extension X-rays, using atlanto dens interval (ADI). ADI of greater than 5 mm was taken as definition of atlantoaxial instability (figure 1).

**Figure 2:**
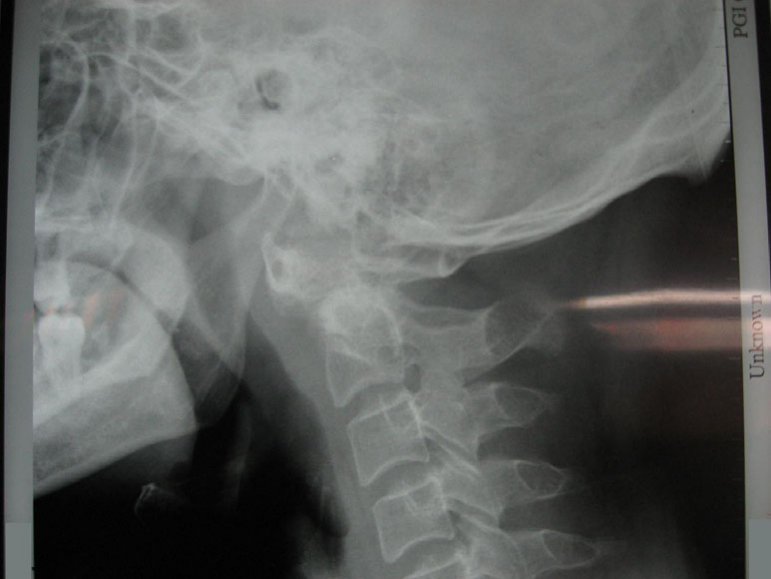
**Post reduction film of the same subject using skeletal traction in the ward**. Further complete reduction was obtained intraoperatively using skeletal traction with crutchfield tongs.

All subjects were assessed with plain anteroposterior, open mouth view and lateral flexion extension radiographs. Lateral radiographs help to verify that the C1-C2 complex has been reduced adequately before the surgery and to find the estimated length of the screws to be used (figure 2).

**Figure 3:**
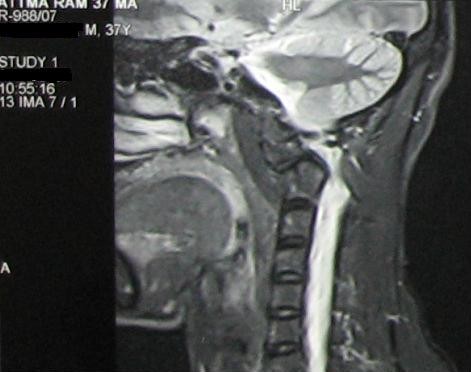
**MRI showing cord compression due to anterior translation of the axis over atlas in a subject with atlantoaxial instability**.

A Computed Tomography Scan with saggital, coronal and 3 D reconstruction was done in all the cases to look at the transverse foramen of C2, understand the fracture anatomy, C2 isthmus size, space available for the cord and integrity of the C1 lateral masses. Magnetic Resonance Imaging (MRI) was done only in subjects with neurological deficit, to study the lesion of the cord and the degree of canal compromise, in order to plan posterior decompression in these cases (figure 3).

Subjects who had pathology of the C1-C2 facets and C1 lateral masses, such as comminuted fractures or the tumors destroying the C1 lateral masses that preclude screw placement were excluded from the study. Subjects who were found to have anomalous course of the vertebral artery on Computed Tomography Scan were also excluded from the study. This was studied using axial and saggital cuts of the CT scan in the region of transverse foramen of the C2 vertebra. High riding vertebral artery was identified as having a too medial and/or a cranial course, recognized by the medial or cephalad location of transverse foramen. This reduces the distance between the spinal canal and the medial wall of the transverse foramen, thereby placing the vertebral artery in the path of the screw. The screw trajectory was taken as neutral to about 15 degrees medial from the starting point at the inferomedial angle of C2-C3 facet.

All traumatic cases were screened for other associated spinal and extraspinal injuries, using clinical examination and necessary investigations. Other preoperative variables that were assessed included risk factors for nonunion, pathological abnormality responsible for C1-C2 instability, subject's clinical status including pain and presenting radiological findings. The neurological status was documented using Frankel's Grades.

We used two transarticular screws for fixation of C1-C2 complex combined with bone grafting. The placement of transarticular screws was similar in technical details to the technique described by Magerl et al in 1979 [1]. We used iliac crest bone graft measuring about 3 × 2 cm harvested from the posterior iliac crest. The lamina of the C2 vertebra and C1 arch were decorticated before application of the bone graft with a high speed burr. C1 C2 facet joints were also curetted to enhance fusion. Bone graft was placed between the posterior arch of C1 and the spinous process of the C2 vertebra. The graft press fits in the space once nibbled to appropriate shape. In subjects where posterior decompression was carried out and laminectomy of the C1 was done (n = 10), this graft could not be placed in the midline. We used morselised bone graft placed along the bilateral facet joints in these cases.

Postoperatively, all subjects were kept in a Philadelphia collar for 6 weeks. The subjects were followed up for pain, fusion rates, neurological status and radiographic outcomes. Initial follow up was at 3 months, then at 6 months and 1 year. Subsequent follow up was done annually. Fusion was defined radiologically as evidence of continuity of trabecular bone formation between C1 and C2 across the graft, without lucency or resorption of the graft or hardware failure. Position of the screws was assessed by transoral, anteroposterior and lateral radiographs. A screw was considered well positioned when both the lateral and anteroposterior projections showed both screws lying entirely within the bone and crossing the joint space in the anteroposterior view. Stability was accepted if there was no change in atlantodens interval during flexion and extension studies. Range of neck motion in rotation was also noted in the follow up.

Final outcome was graded both subjectively and objectively, using the scoring system given by Grob et al [6]. Subjectively, the results were graded as good (no serious pain, no restriction of activity); fair (periods of pain, working capacity reduced); or bad (permanent severe pain and disability). The objective rating was good (no pain, solid fusion); fair (moderate pain, solid fusion); or bad (nonunion with severe pain) [15].

## Results

A total of 38 subjects were studied. Of them 29 were males (76%) and 9 were females. The mean age at the time of surgery was 35 years (range 9 to 63 years). Trauma was the most common cause of atlantoaxial instability, seen in 34 (89.5%) subjects. Most common mode of trauma was road traffic accident, in 29 of these 34 subjects. The distribution of subjects by etiology is given in table 1. All subjects with traumatic atlantoaxial instability had fracture of the odontoid process. Type II D' Olanzo fracture was seen in 30 of these subjects. In 4 subjects it was type III fracture. Indications for arthrodesis in these subjects with odontoid fracture were established nonunion or age more than 60 years. There were five cases of non union of odontoid fracture secondary to failed anterior screw fixation for the fracture of the odontoid. They were operated after mean of 7 months after injury. In 8 subjects the initial injury to the upper cervical spine was missed at their initial referral center. These subjects presented late with neck pain and stiffness at 3-6 months from injury. In 21 subjects, fracture odontoid was managed conservatively at their initial referral centre with immobilization or traction. There were three cases with rheumatoid arthritis. Mean ADI in these cases was 10.5 mm. All these subjects had neurological deficit.

**Table 1:** Etiology of atlantoaxial instability

Etiology	Frequency	Percentage
trauma	34	89.5%
rheumatoid arthritis	3	8%
tuberculosis	1	2.5%
**Total**	**38**	**100%**

Most common presenting symptom was neck pain, present in 35 of the 38 subjects (92%) in our series. Neurological deficit was present in 17 subjects (44.7%). Of these 15 subjects had quadriparesis and 2 subjects had monoplegia. Out of the 34 traumatic cases 14 had neurological deficit. All 3 subjects with rheumatoid arthritis had neurological deficit. Worsening of neurological deficit over time was seen in 3 subjects. Two of these subjects had rheumatoid arthritis, and the third had history of road side accident.

The most common risk factor for nonunion in our subjects was smoking, seen in 8 subjects (21%). The other factors included-rheumatoid arthritis, in 3 subjects; steroid intake in 3 subjects and diabetes mellitus in 3 subjects.

**Figure 4:**
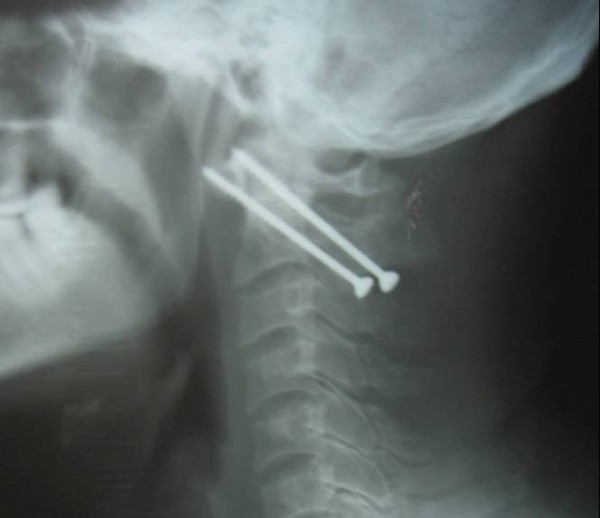
**Postoperative radiograph of the subject showing placement of two transarticular screws across the reduced atlantoaxial joints**.

**Figure 5:**
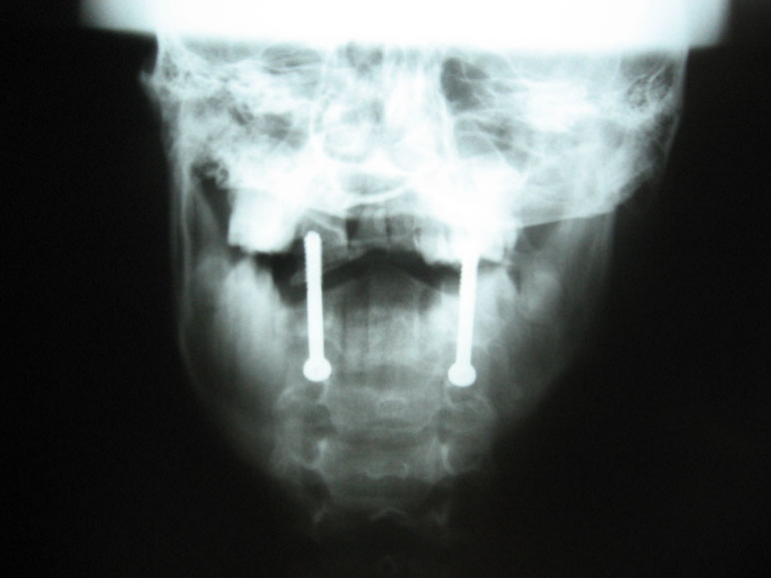
**Anteroposterior open mouth view showing placement of transarticular screws**.

Postoperative radiographs showed adequate reduction of C1 over C2 in 35 cases. Adequate screw placement was seen in 31 cases (figure 4 &5). In one patient only one screw could be placed due to vertebral artery injury on that side. Another subject had screw cutout. She was a case of rheumatoid arthritis, and was taking steroids for a long period. Radiographs were suggestive of markedly reduced bone density. She did not progressed to union, and neurological deficit persisted in her. In the third patient the screw placement was a little too lateral and superior, and the screws penetrated out of the anterior cortex of the anterior arch of C1. The future course was uneventful in this patient. The mean screw length was 42 mm.

Posterior decompression was combined with the procedure in 10 subjects with neurological deficit and evidence of cord compression on Magnetic Resonance Imaging (MRI). All 3 cases with rheumatoid arthritis had undergone posterior decompression. Vertebral artery injury was seen in 3 subjects. None of these subjects had anomalous transverse foramen or abnormally narrow isthmus on preoperative Computed Tomography (CT) scan. When vertebral artery injury was encountered intraoperatively, screw was placed in the drill hole to provide a temponade effect. Placement of screw on the other side was not attempted for the fear of injuring both the vertebral arteries. In one of these subjects only one screw could be placed since the artery was hit on the side being operated first. In none of these subjects any sign of neurological deficit or vertebral insufficiency was seen, probably because of sufficient collateral circulation [16,17].

Fusion was seen in 35 cases. In three cases the graft showed resorption, and there was no evidence of formation of bony bridge between C1 and C2. Earliest radiological evidence of union could be seen in these patients at a mean follow up of 3.6 months. There was no instance of deep infection of the surgical site or the graft site. Decubitus ulcers on the occiput were seen in two subjects. Suboccipital paresthesia and numbness was present in 3 patients.

The most common postoperative complaint was residual neck and occipital pain, seen in 8 subjects. At the latest follow up, subjectively, 24 subjects had good result, 6 had fair and 8 had bad result. On objective grading, 24 had good result, 11 had fair and 3 had bad result. The mean follow up duration was 41 months (range 15-70 months).

At admission 17 subjects had neurological deficit. Of these 14 were Frankel's grade C and 3 were Frankel's grade D. At discharge 10 subjects had completely recovered, with neurological deficit persisting in only 7 subjects. All these 7 subjects belonged to Frankel's grade C.

The mean range of neck motion was 40 degrees of lateral rotation on left side and 35 degrees of rotation on right side. Since atlantoaxial fusion virtually eliminates the motion at C1-C2 joint, the residual rotation reflects the subaxial component of the neck motion. This range of motion was maintained on follow up. The mean range of lateral rotation in cases of rheumatoid arthritis was 25 degrees on each side. This is consistent with the view that rheumatoid spine has restricted range of atlantoaxial and subaxial motion. The range of flexion and extension was maintained after surgery. The mean flexion and extension arc was 150 degrees. The mean range of lateral bending was 45 degrees on each side.

## Discussion

The aim of treatment of atlantoaxial instability is to achieve a solid fusion between C1 and C2, virtually eliminating any motion between them. This is expected to relieve the neck pain and avoid the risk of further neurological deficit. The posterior wiring techniques popularized by Gallie et al [18] and Brooks and Jenkins [19] had been the most common means of stabilization in the past. In recent years, a variety of other techniques have been used, such as, interlaminar clamps, polyaxial screw and rod fixation, transarticular screw fixation and C1 lateral mass screws with C2 pars screw fixation. Posterior C1-C2 transarticular screw fixation has become the gold standard for atlantoaxial fusion. It has lead to considerable improvement in the fusion rates upto more than 95% [1-6] over C1-C2 wiring procedures, whose failure rates range from 10% to 25% [20,21]. Taggard et al [7] conducted a case control study to compare the fusion rates using transarticular screws and posterior wiring techniques. After a mean follow up of 31 months they found that successful fusion was achieved in 13 of 14 subjects treated with the TAS technique as compared to 5 out of 13 subjects who underwent a posterior wiring technique. They observed that subjects with a radiographically solid fusion were 21 more times likely to have undergone TAS than posterior wiring technique (p = 0.004). The position of the transarticular screws is closer to the centre of axis of rotation and lateral bending, which provides better control of movements than other techniques which rely on peripheral fixation.

The biomechanics of surgical stabilization of the C1-C2 articulation can be divided into three different types. One-point fixation stabilizes the motion segment only posteriorly (e.g. Gallie wiring, Halifax clamps etc). Two-point fixation construct includes transarticular screws through the laterally placed facet joints. Three-point fixation consists of the combination of the two previous principles, thus stabilizing the C1-C2 motion segment both laterally and posteriorly. In biomechanical testing three point fixation has been found to be superior to both two-point and one-point fixations [22-26]. Thus the tension band construct provides two advantages-first, it enhances the stability of the TAS fixation; and second, the structural bone graft is stabilized by the wire. But sublaminar wire passage carries the potential risk of neurological complications [9-11], especially in cases where the canal has already been compromised. Further this wire-graft technique is technically demanding and time consuming [2,27]. Some reports have shown that metal wires or cables may bow anteriorly because of "spring phenomenon" even without any breakage, leading to encroachment upon the spinal cord [13,28].

It is controversial in literature whether posterior wiring construct provide any additional contribution towards fusion. Matsumoto *et al *reported 18 cases of loosening of posterior wiring construct in 52 cases with 95% fusion rate [14]. In Ito's series, all cases had loosening, but with 100% fusion rate. Thus, wire or cable loosening did not lead to nonunion or pseudarthrosis, but it might endanger the spinal cord. From these observations, Ito et al came to the conclusion that adding wire construct is not required [13]. Avoiding the placement of posterior wires may be especially important in situations where inflammatory disease with soft tissue swelling and pannus has resulted in compromise of the spinal canal, or in the case of C1-C2 subluxation which is not completely reducible [8]. Significant degenerative changes or osteoporosis of the posterior elements of C1 and C2 also preclude the use of posterior wiring techniques. Wang et al [4] achieved solid fusion in all their 57 subjects, using only morselized autograft and transarticular screw, without any posterior wiring construct. We did not use the morselized graft but a strut of iliac crest graft well fitted in the space between the C1 lamina and C2 spinous process. Thus, although from the biomechanical viewpoint, bilateral TAS fixation may not be as stable as the 3-point fixations, fusion rates have not been altered. There is only slight micromotion left in flexion-extension after fixation. We supposed that this micromotion would not affect fusion. In our series, there is no loss of the reduction and the fusion rate is 92%. This is in unison with the fusion rates achieved by other authors who used combination of Transarticular screws and posterior wiring [1-7]. Randomized or a case control study will be a better study design to study this effect. But correspondence of our results with those of studies using Magerl's fixation suggests that this technique is a sound alternative thus simplifying the Magerl's technique.

Though single screw placement is expected to lead to nonunion, there is no convincing data in this regard. In our study single screw was placed in 1 subject. Solid union was achieved in this subject at follow up. Song et al [23] concluded that unilateral C1-C2 transarticular screw fixation with interspinous bone graft wiring is an excellent alternative in the treatment of atlantoaxial instability when bilateral screw fixation is contraindicated. They reported a solid fusion using this technique in 18 of 19 subjects with atlantoaxial instability and unilateral anomalies. Grob et al [6] found that nonunion did not follow incorrect placement of one screw, so bilateral fixation is not an indispensable condition for a satisfactory outcome.

Posterior transarticular screw fixation has several advantages over other fixation techniques. Contrary to the traditional posterior fusion techniques, the integrity of the ring of C1 is not necessary for transarticular screw placement. Thus this technique can be used even in cases of fracture or the absence of posterior arch of the atlas. This technique also provides approach for laminectomy, if needed for decompression of the cord. Further, there is no implant inside the spinal canal as in the wiring techniques and complications associated with wire loosening are avoided. A very important advantage is that it avoids the need for postoperative halo immobilization, when compared to the posterior wiring techniques. This is an important factor from the subjects' point of view for the selection of the procedure. Achieving preoperative reduction is imperative for safe atlantoaxial fusion. Displacement of C1 on C2 decreases the space available for the cord. This distorts the C1 C2 alignment, and the placement of transarticular screws is not completely safe. This also increases the risk with sublaminar wire passage, because of increased chances of hitting the cord. Although some authors have used transarticular screw fixation for in situ fixation, the precise limit beyond which this technique is contraindicated is not defined. Thus in large fixed displacements of C1 on C2, occipitocervical fusion with C1 decompression, or anterior decompression and fusion are indicated [8].

The disadvantages of this procedure include need for an extensive skin incision and soft tissue dissection to expose the entire dorsum of C2. This extensive posterior exposure has been associated with a complication rate as high as 10%, including superficial infections and occipital nerve injury [8,29]. Screw placement requires an acute angle for proper screw trajectory, which may be impeded by kyphotic deformities or by moving the neck anteriorly. Additionally, there is a steep learning curve for this technique. Complications associated with this technique include the potential for vertebral artery injury, malposition of screws, pseudoarthrosis, implant failure, dural tear, hypoglossal paresis, brain stem infarction and death. Inconstant size and location of the transverse foramen in the lateral mass of the axis places the vertebral artery at risk during drilling and screw placement. Scans with saggital and coronal reconstructions help to assess the relationship of transverse foramen of C2 and the C1-C2 facet joint to determine the correct trajectory for the screw and avoid arterial injury [30,31]. Radiographic and anatomical studies of the atlanto-axial complex suggest that upto 20% of the subjects have atlanto axial anatomy that precludes safe bilateral screw placement [32-34]. We had 3 cases of vertebral artery injury in our study (8%). Reported rates of vertebral artery injury using this technique vary from 0-10% in different series [17,8,29,32,35-39]. American Association of Neurological Surgeons/Congress of Neurological Surgeons (AANS/CNS) Section on disorders of Spinal Nerves and Peripheral Nerves in their survey published by Wright and Lauryssen [35], estimated the risk of vertebral artery injury during C1-C2 transarticular screw fixation to be 2.2% per screw inserted. The risk of neurological deficit from vertebral artery injury was 0.2% per subject, and the mortality rate was 0.1%. Thus injury to vertebral artery is well tolerated in the majority of the subjects. Despite numerous reports of vertebral artery injuries, resultant neurological deficit is rare [8]. Coric D et al [40] reported a case of vertebral artery to epidural venous plexus fistula as a complication of posterior atlantoaxial facet screw fixation. Madawi et al [33] reported five cases of vertebral artery injury (8.2%) in subjects who underwent this operation. He also pointed out that incomplete reduction is a risk factor for inadequate screw placement. Incidence of dural tears has been reported to be 0.3%. suboccipital numbness is relatively common, seen in 16.8% patients in report by Wright and Lauryssen [35]. In most of them however it resolved spontaneously with time.

Despite excluding all the patients with dangerous anatomy of the vertebral artery, we still had 3 patients in whom vertebral artery injury was observed. Two of these patients were observed in the first half of the study period when the experience of the surgeon with this technique was relatively recent. This is a highly surgeon dependent technique and learning curve is high. Surgeon has to be familiar with the anatomy of the transverse foramen in the upper cervical spine. This needs experience with studying a large number of CT scans. Failure to meticulously identify the danger in this region may lead to catastrophy.

The studies of RA subjects showed relatively lower rates of bony union than did the studies with smaller percentages of RA subjects [29,41-43]. Literature suggests that presence of rheumatoid arthritis entails the risk of posterior graft nonunion more than other disorders [6,41-43]. We achieved union in only of the 3 patients with RA. Due to the small sample size with only 3 subjects with rheumatoid arthritis, no statistically significant conclusion regarding effect of rheumatoid disease on fixation and union can be reached. Ito T et al found that in 5 of their 7 subjects with rheumatoid arthritis who had nonunion, C1-C2 complex was stable due to fusion at the facet joints, as demonstrated by functional radiographs and computed tomography scans [13]. Thus atlantoaxial transarticular screws can bring the facet fusion despite the posterior graft failure in such cases.

## Conclusions

Thus, transarticular screw fixation is an effective technique for the fusion of the atlantoaxial complex. It provides highest fusion rates, and is particularly important in subjects at risk for nonunion. It has expanded the indications for atlantoaxial fusion and is an important salvage technique in subjects with previous failed procedures. Although its learning curve may be steep, it is associated with few rates of complications in expert hands.

## Competing interests

The authors declare that they have no competing interests.

## Authors' contributions

RB is the senior authors who carried out the surgical procedure, coordinated the planning of preoperative and postoperative protocols, and helped to draft the manuscript. TG had the instrumental role in the planning and execution of perioperative and intraoperative design of the study and preparation of the manuscript. SSD and ST helped in acquisition of data and in drafting of the manuscript. All authors read and approved the final manuscript.
